# Deducing the Conformational Properties of a Tyrosine Kinase Inhibitor in Solution by Optical Spectroscopy and Computational Chemistry

**DOI:** 10.3389/fchem.2020.00596

**Published:** 2020-07-28

**Authors:** Md. Lutful Kabir, Frederick Backler, Andrew H. A. Clayton, Feng Wang

**Affiliations:** ^1^Department of Physics and Astronomy, Optical Sciences Centre, Faculty of Science, Engineering and Technology, Swinburne University of Technology, Melbourne, VIC, Australia; ^2^Department of Chemistry and Biotechnology, Centre for Translatonal Atomaterials, Faculty of Science, Engineering and Technology, Swinburne University of Technology, Melbourne, VIC, Australia

**Keywords:** tyrosine kinase inhibitor (EGFR-TKI), drug structures, density functional theory (DFT) calculations, spectroscopy, off-target

## Abstract

Dacomitinib (PF-00299804) was recently approved by the Food and Drug Administration (FDA) as a tyrosine kinase inhibitor (TKI). Unfortunately, side effects and disease resistance eventually result from its use. Off-target effects in some kinase inhibitors have arisen from drug conformational plasticity; however, the conformational states of Dacomitinib in solution are presently unknown. To fill this gap, we have used computational chemistry to explore optimized molecular geometry, properties, and ultraviolet-visible (UV-Vis) absorption spectra of Dacomitinib in dimethyl sulfoxide (DMSO) solution. Potential energy scans led to the discovery of two planar and two twisted conformers of Dacomitinib. The simulated UV-Vis spectral signatures of the planar conformers reproduced the two experimental spectral bands at 275 and 343 nm in solution. It was further discovered that Dacomitinib forms conformers through its three flexible linkers of two C–NH–C bridges, which control the orientations of the 3-chloro-4-fluoroaniline ring (Ring C) and the quinazoline ring (Rings A and B) and the 4-piperidin-1-yl-buten-2-nal side chain, and one C–O–C local bridge which controls the methoxy group locally. When in isolation, these flexible linkers form close hexagon and pentagon loops through strong intramolecular hydrogen bonding so that the “planar” conformers Daco-P1 and Daco-P2 are more stable in isolation. Such flexibility of the ligand and its ability to dock and bind with protein also depend on their interaction with the environment, in addition to their energy and spectra in isolation. However, an accurate quantum mechanical study on drug/ligand conformers in isolation provides necessary reference information for the ability to form a complex with proteins.

## Introduction

Lung cancer is the most lethal malignancy among all kinds of cancer worldwide, and non-small-cell lung carcinoma (NSCLC) is counted as the most prevalent (Abdelhameed et al., 2019; Lau et al., 2019). Overexpression of epidermal growth factor receptors (EGFR) and abnormal signaling through the human epidermal growth factor receptor family (HERs) are considered as contributors to NSCLC (Huang et al., 2009; Costa and Kobayashi, 2015). Quinazoline derivatives designed to block HER activation are popular antitumor agents (Selvam and Kumar, 2011; Shagufta, 2017; Qiu et al., 2019; Solyanik, 2019). Dacomitinib (Reed and Smaill, 2016) (trade name: Vizimpro), an anilinoquinazoline derivative, recently received FDA approval (Shirley, 2018; Roskoski, 2019) as a tyrosine kinase inhibitor (TKI) for the treatment of locally advanced or metastatic NSCLC, as it irreversibly inhibits three out of four HER families: HER-1, HER-2, and HER-4 tyrosine kinases (Gonzales et al., 2008). This highly selective, small-molecule TKI of the epidermal growth factor receptor (EGFR) (Bello et al., 2013) has the aniline–quinazoline ring structures with an acrylamide branch known as the Michael acceptor. This Michael acceptor, a chemically reactive electrophilic warhead, targets a cysteine nucleophile in the adenine pocket to form an irreversible covalent adduct (Gajiwala et al., 2013).

Despite high controversy (Caruso, 2018; Passaro and de Marinis, 2018) and earlier recommendations for use in second-line treatment (Gonzales et al., 2008; Zugazagoitia et al., 2017), dacomitinib was recently suggested as a first-line clinical therapy worldwide (Wu et al., 2017; Decoster et al., 2018; Shirley, 2018; Abdelhameed et al., 2019; Chustecka, 2019; Roeper and Griesinger, 2019). In addition, this more potent TKI could possibly find other applications such as suppressing brain tumor (Chen et al., 2019), chemoresistant ovarian cancer (Momeny et al., 2017), and breast cancer (Kalous et al., 2012) in future.

Compared to the first-generation anticancer agents, Dacomitinib has prolonged progression-free survival and overcame the resistance from the EGFR-muted NSCLC (Gonzales et al., 2008; Lau et al., 2019) but at the expense of increased cytotoxicity (Zugazagoitia et al., 2017). Still, NSCLC eventually acquired resistance to Dacomitinib by either EGFR T790M or C797S mutations (Kobayashi et al., 2018). Commonly found adverse reactions of this EGFR antagonist are diarrhea, rash, nail changes (paronychia and onycholysis), fatigue, stomatitis, loss of appetite, conjunctivitis, weight loss, alopecia, cough, itching, nausea, etc. (Shirley, 2018; Chustecka, 2019). These side effects may possibly result from its two reactive metabolites (Attwa et al., 2018). Alternatively, unexpected off-target effects can also arise from drug conformational plasticity (Hantschel, 2015), i.e., distinct drug conformers, which bind on-target and off-target proteins. It is therefore important to determine the relevant conformational states in solution.

**Scheme 1:**
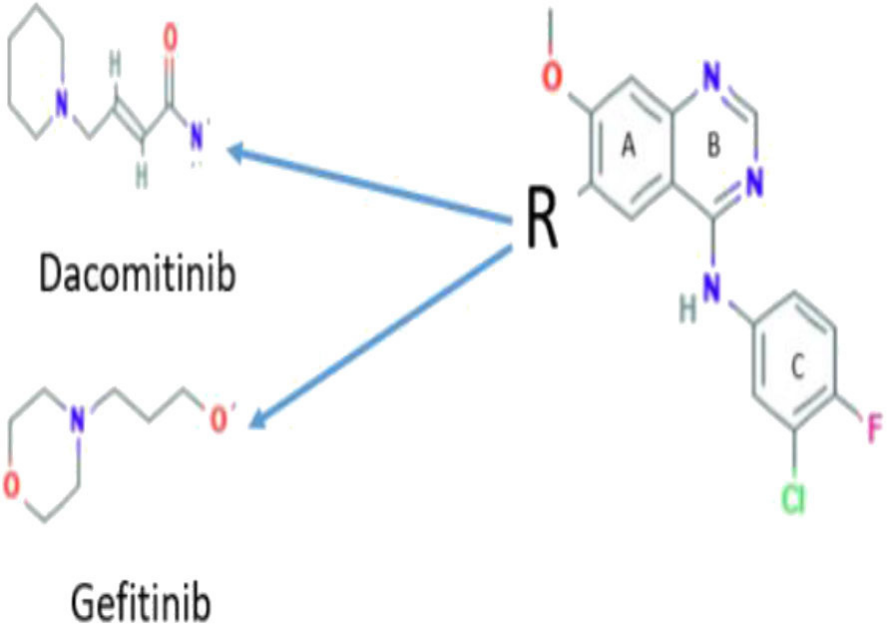
Chemical structures of Dacomitinib and Gefitinib and their relationship.

As Dacomitinib is a very newly FDA-approved drug (Shirley, 2018; Roskoski, 2019), to the best of our knowledge, there are no reported detailed electronic structural studies of the drug and its conformation in solution, although the crystal structure of the drug is available and drug conformation in the drug–protein complex (Gajiwala et al., 2013) was also reported. Without detailed molecular structural information and knowledge about how the drug responds to the environment, it is difficult to understand the mechanism of the ligand–protein interaction. The mechanism of the ligand–protein interaction is important for the development of new drugs. For example, Dacomitinib (PF00299804) is considered in the same class of a NSCLC drug Gefitinib (ZD1839) as shown in Scheme 1, which differs only in the side chain. However, Dacomitinib (PF00299804) and Gefitinib (DZ1839) exhibit significant drug potent activities. The former effectively inhibits the *in vitro* kinase activity of wild-type *EGFR* (IC50 = 6 nM) (Engelman et al., 2007), whereas the latter is an inhibitor that specifically binds and inhibits the EGFR tyrosine kinase, with the IC_50_ value of 2–37 nM in NR6wtEGFR cells (Pedersen et al., 2005).

In order to understand how the chemical structural changes in Gefitinib and Dacomitinib affect the drug potency and ability to bind with proteins, conformation(s) in solution is required. The present study provides the first accurate quantum mechanical electronic structures and conformations of Dacomitinib in solution. In this study, the ultraviolet-visible (UV-Vis) absorption of Dacomitinib in dimethyl-sulfoxide (DMSO) solution and *in silico*, for the first time, is investigated applying the time-dependent density functional theory (TD-DFT). The experimental measurements together with computational calculations elucidate the interrelationship of the molecular structure and spectral properties of Dacomitinib.

## Materials and Methods

### Experimental Details

Dacomitinib and DMSO were purchased from Focus Bioscience Pty Ltd. (MedChemExpress) and Sigma-Aldrich Pty Ltd., respectively. All UV-Vis absorption experiments were carried out on the same day, using a pair of matched quartz cuvettes (purchased from Stama Pty Ltd.) with a 1-cm path length. The electronic absorption spectra (absorbance of λ_max_ < 0.1) of the Dacomitinib solution in DMSO (5–25 μL) in the range of 250–400 nm was recorded on a Perkin-Elmer LAMBDA 1050 UV/Vis/NIR spectrophotometer at room temperature (293 K).

### Computational Details

**Figure 1:**
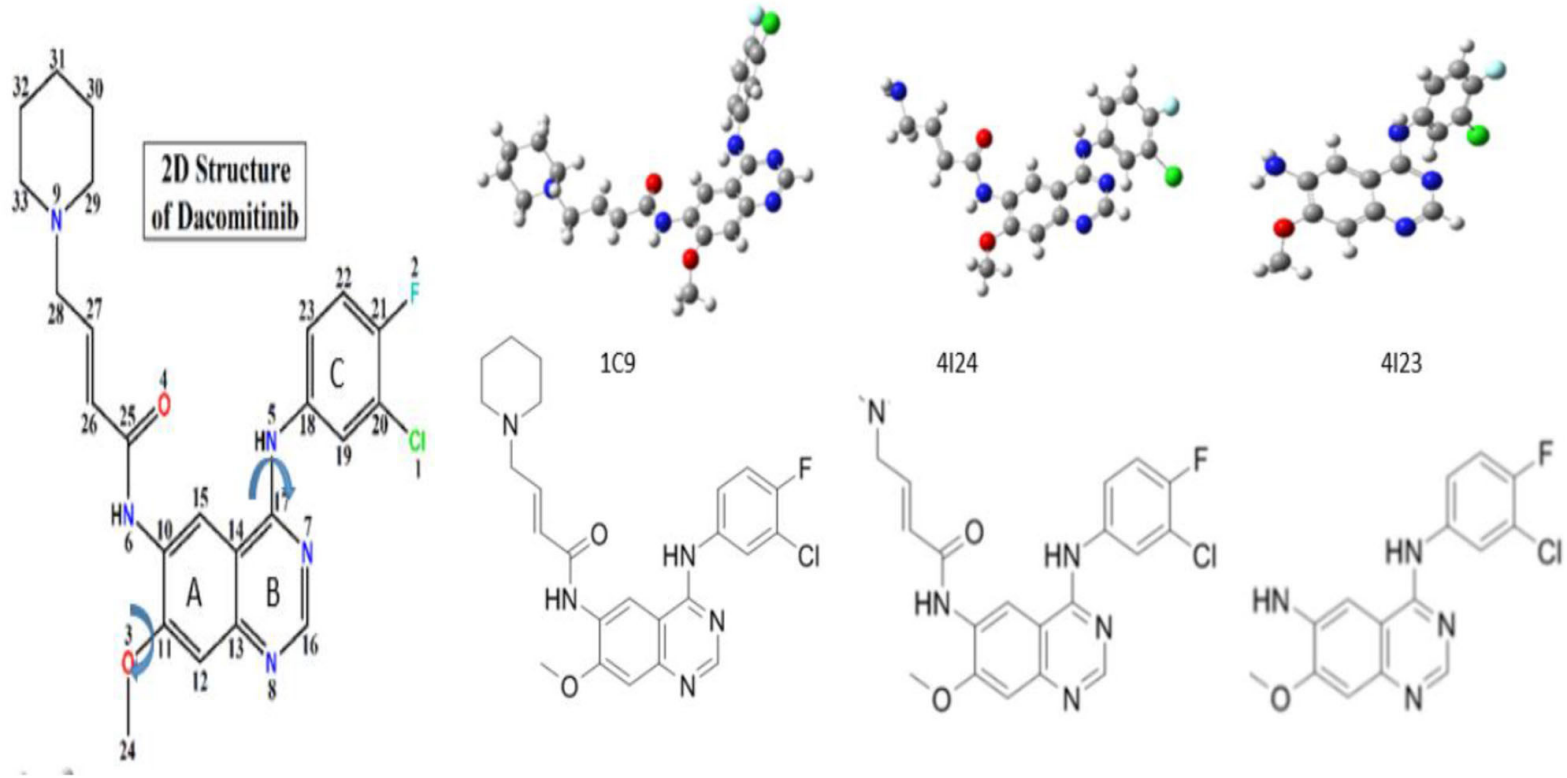
Nomenclature and chemical structure of Dacomitinib ((2E)-N-(4-((3-chloro-4-fluorophenyl)amino)-7-methoxyquinazolin-6-yl)-4-piperidin-1-ylbut-2- enamide). The Arrows indicate the most likely rotations. The crystal structure of (1C9) (Lau et al., 2019) and the co-crystallized structures of Dacomitinib from the Protein Data Bank (PDB), 4I23 and 4I24 (Abdelhameed et al., 2019). The dihedral angles of C(12)-C(11)-O(3)-C(24) and C(19)-C(18)-N(5)-C(17) define the flexibility of the ligand structures, which vary according to the conditions of crystallization of the drug (ligand).

The IUPAC name of Dacomitinib (C_24_H_25_ClFN_5_O_2_) is (2E)-N-{4-[(3-chloro-4-fluorophenyl)amino]-7-methoxyquinazolin-6-yl}-4-(piperidin-1-yl)but-2-en amide. The initial 3D geometry of the crystal structure of Dacomitinib was downloaded from the PubChem website (https://pubchem.ncbi.nlm.nih.gov/compound/Dacomitinib#section=3D-Conformer) with a ligand ID code of 1C9 in Figure 1. Optimization of this moiety in DMSO was performed using the density-functional theory (DFT)-based Becke three-parameter Lee–Yang–Parr hybrid functional (B3LYP) (Becke, 1993) in combination with the 6-311+G(d,p) basis set and the conductor-like polarizable continuum model (CPCM) (Cossi et al., 2003). Potential energy scan (PES) was performed by relaxed scan through the C_(17)_-N_(5)_ bond using B3LYP/6-311G. The resulting local minima geometries were further optimized at B3LYP/6-311+G(d,p) in the DMSO solvent. Absorption UV-Vis spectra in DMSO solution were calculated using the time-dependent (TD)-DFT method for the lowest 40 excited states of singlet–singlet transitions using the same model. The UV-Vis spectrum of the most stable structure of the drug Daco-P1 in DMSO solution was calculated using both B3LYP/6-311+G(d,p) and CAM-B3LYP/6-311+G(d,p) methods, respectively. The method has been established in our previous studies of this class of TKIs such as AG-1478 (Khattab et al., 2016) and SKF86002 (Van Dongen et al., 2017). The excess orbital energy spectrum (EOES) proposed by Islam and Wang (2015) was employed to examine the core electron energies between the Dacomitinib conformers. The EOES recognizes which orbital sites change in energy in response to structural changes through conformation (Wang and Chatterjee, 2017); EOES was performed by analyzing the orbital energies of the higher-energy conformers with respect to those of the global minimum structure (Khattab et al., 2016). All calculations were carried out using the Gaussian 16 Revision A.03 computational chemistry package (Frisch, 2016).

## Results and Discussion

### Dacomitinib Conformers in Crystal Phase and in Isolation

The chemical structure and nomenclature of Dacomitinib (C_24_H_25_ClFN_5_O_2_, CAS 1110813-31-4) are given in Figure 1. A single-bond NH linker, C_(17)_-N_(5)_(H)-C_(18)_, connects the 3-chloro-4-fluoroaniline ring (Ring C) and the quinazoline ring (Rings A and B). The initial crystal structure of Dacomitinib was downloaded from the PubChem website (https://pubchem.ncbi.nlm.nih.gov/compound/Dacomitinib#section=3D-Conformer) with a ligand ID code of 1C9. The Dacomitinib co-crystal structures by Gajiwala et al. (2013) with IDs of 4I23 and 4I24 were downloaded from the Protein Data Bank (PDB) for more structural information. The three crystal structures of Dacomitinib are given in Figure 1. The co-crystal ligand structures (4I23 and 4I24) are not the complete chemical structure of Dacomitinib but are the core structure of 3-chloro-4-fluoroaniline ring and the quinazoline ring present in 4I24 (without piperidin-1-yl) and in 4I23 (without 4-piperidin-1-yl-buten-2-nal). As can be seen in this figure, the conformations of the Dacomitinib ligand depend on both the crystallization conditions and the complexes that the ligand forms with the proteins (polymers). The most apparent conformational changes are in three major bridges of C_(10)_-N_(6)_H-C_(25)_, C_(11)_-O_(3)_-C_(24)_ and C_(17)_-N_(5)_-C_(18)_, which control the flexibility of the ligand (drug molecule) through intramolecular hydrogen bonding (HB). The two N-bridges control the relevant orientations of the 3-4-piperidin-1-yl-buten-2-nal tail and the chloro-4-fluoroaniline ring (C) with respect to the quinazoline ring (Rings A and B). The O-bridge provides small tunes to the methoxy group (-OCH_3_) and the quinazoline ring (Rings A and B) locally.

The core structure of Dacomitinib is the two-ring backbone system [3-chloro-4-fluoroaniline ring (Ring C)] and the quinazoline ring (Rings A and B) as shown in Scheme 1. Variations of the side R-chain and results in Gefitinib and Dacomitinib are shown in Scheme 1. The crystal structures (1C9, 4I24, and 4I23) reported in Figure 1 have different side R-chains, and only the 1C9 structure contains the full Dacomitinib ligand structure (piperidin-1-yl is missing in 4I24 and 4-piperidin-1-yl-buten-2-nal is missing in 4I23). Table 1 collects the selected structural properties of the ligand in Figure 1 from different sources, that is, the same ligand when being confined in crystal or co-crystal complexes is different from that in isolation (solution may be considered in this case). Moreover, under different crystallization conditions, the ligand results in different conformations with respect to the flexible regions.

**Table 1:** Selected geometric properties of crystal structure (literature) and calculated Dacomitinib conformers obtained.

**Selected properties**	**In crystal (database)**			In isolation (calculated^*^)			
	**Crystal struct**	**4I24 PDB**	**4I23 PDB**	**Daco-P1**	**Daco-P2**	**Daco-T1**	**Daco-T2**
R_A_/Å	8.363	8.571	8.588	8.375	8.375	8.382	8.382
R_B_/Å	8.179	8.302	8.348	8.160	8.161	8.158	8.157
R_C_/Å	8.376	8.519	8.550	8.302	8.303	8.295	8.293
C-F/Å	1.341	1.341	1.344	1.341	1.341	1.339	1.339
C-Cl/Å	1.722	1.722	1.731	1.735	1.733	1.733	1.732
C(18)-N(5)/Å	1.404	1.418	1.423	1.398	1.399	1.411	1.416
C(17)-N(5)/Å	1.417	1.431	1.441	1.359	1.359	1.366	1.366
C(10)-N(6)/Å	1.417	1.424	1.415	1.394	1.394	1.394	1.395
C(25)-N(6)/Å	1.389	1.405	–	1.362	1.362	1.363	1.362
C(28)-N(9)/Å	1.463	1.509	–	1.451	1.451	1.451	1.451
C(15)H···O(4)/Å	2.107	2.815	–	2.142	2.140	2.152	2.159
N(6)H···O(3)/Å	2.391	2.078	2.279	2.092	2.093	2.100	2.097
C(19)H···N(7)/Å	2.772	2.605	2.827	2.207	2.228	2.324	4.793
C(11)-O(3)-C(24)/°	116.99	120.02	118.10	118.60	118.60	118.63	118.64
C(17)-N(5)-C(18)/°	132.44	126.79	126.64	131.30	131.35	128.57	126.53
C(10)-N(6)-C(25)/°	127.34	121.78	–	128.35	128.33	128.12	128.31
C(20)-C(21)-F/°	120.70	120.86	120.19	120.10	119.80	119.64	119.63
C(21)-C(20)-Cl/°	120.21	121.62	121.42	119.53	119.86	119.73	119.78
C(12)-C(11)-O(3)-C(24)/°	−90.02	−0.654	−31.95	0.225	0.239	0.278	−0.489
C(19)-C(18)-N(5)-C(17)/°	−30.05	28.478	43.97	0.033	178.45	33.547	142.85
RMSD^a^	**–**	**–**	**–**	**0.619**	**0.617**	**1.144**	**1.631**
μ/D	11.967	–	–	12.721	11.132	6.328	7.777
ΔE/kcal·mol^−1^	91.15	–	–	0.0^b^	0.51	4.44	4.60

* Using the B3LYP/6-311+G^**^ model.

a *Root mean square deviation (RMSD) not including the dihedral angles*.

b *The total calculated energy of Dacomitinib in DMSO solution is −1914.271464 E_h_*.

*Grey color indicates hydrogen bonding of the structures; Green color indicates the most important dihedral angles*.

**Figure 2:**
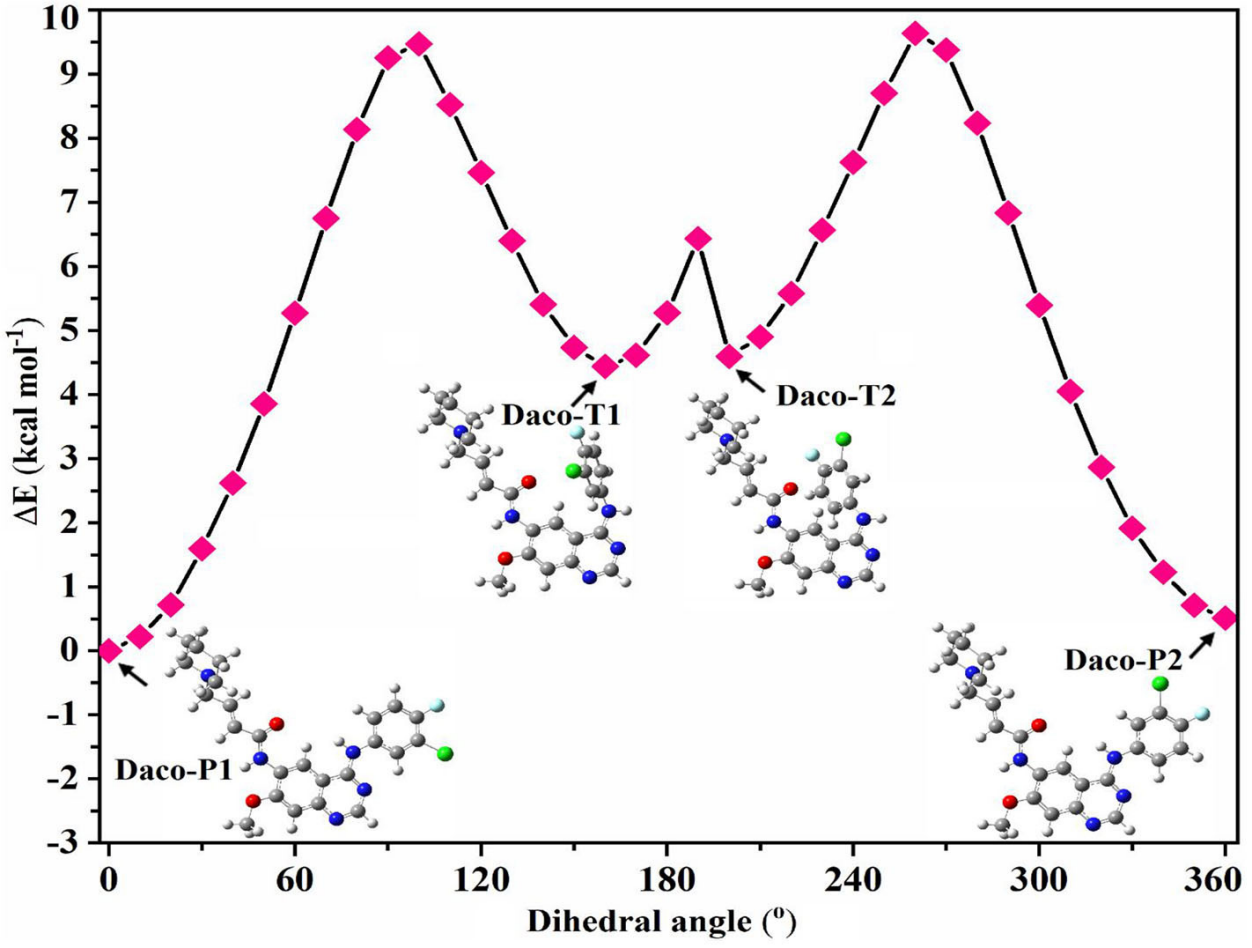
Potential energy surface (PES) scan of Dacomitinib conformers in DMSO through local rotation of the C_(17)_-N_(5)_ bond (dihedral angle ∠N_(7)_-C_(17)_-N_(5)_-H) using the B3LYP/6-311G level of theory. Pointed arrows at a particular point on PES refer to the corresponding 3D structures of Dacomitinib. In the 3D structures of Dacomitinib conformers, atoms of carbon, hydrogen, nitrogen, oxygen, chlorine, and fluorine are denoted by gray-, white-, blue-, red-, green-, and cyan-colored atoms, respectively.

Full geometry optimization was performed to locate a planar minimum energy structure of Dacomitinib (Daco-P1). Here, the “planar” structure Daco-P1 only means that the dihedral angle of the plane formed by Ring C (3-chloro-4-fluoroaniline ring) and Rings A and B (the quinazoline ring) is very small when the drug molecule is in isolation. Based on the obtained structure (Daco-P1), three more local minima (conformers) were found by a potential energy scan (PES) calculation through rotation of the C_(17)_-N_(5)_ bond (i.e., dihedral angle ∠N_(7)_-C_(17)_-N_(5)_-H), which evidently alter neither any bond lengths nor most of the bond angles outside of the selected parameters. The perimeters of the three aromatic rings in four conformers, for example, are altered by <0.01 Å. Figure 2 presents the PES and the obtained local minima and their structures, in which the differences of the bond angles related to the NH linker are negligible too, except that the NH linker local to dihedral angles altered considerably [all include N_(5)_]. In addition, our previous study on a TKI drug AG-1478 (Khattab et al., 2016) in the same class discovered that rotation of the C_(17)_-N_(5)_ bond produced possibly potent conformers of the drug. Therefore, the present study concentrates on the rotation of the C_(17)_-N_(5)_ bond for conformer searches. As an illustration, the dihedral angle of ∠N_(7)_-C_(17)_-N_(5)_-H showed substantial conformational alterations from the planar structure of Daco-P1 to the twisted structure of Daco-T1 by 160°. The obtained four stable local minima (conformers) are in two pairs of “planar” Daco-P1 and Daco-P2 and twisted Daco-T1 and Daco-T2 conformers. A small energy barrier of ~1.5 kcal·mol^−1^ between Daco-T1 and Daco-T2 is as shown in Figure 2. However, for Dacomitinib conformers (Daco-P1 and Daco-P2) to become more stable, the twisted conformers need to cross a higher-energy barrier of ~5 kcal·mol^−1^.

**Figure 3:**
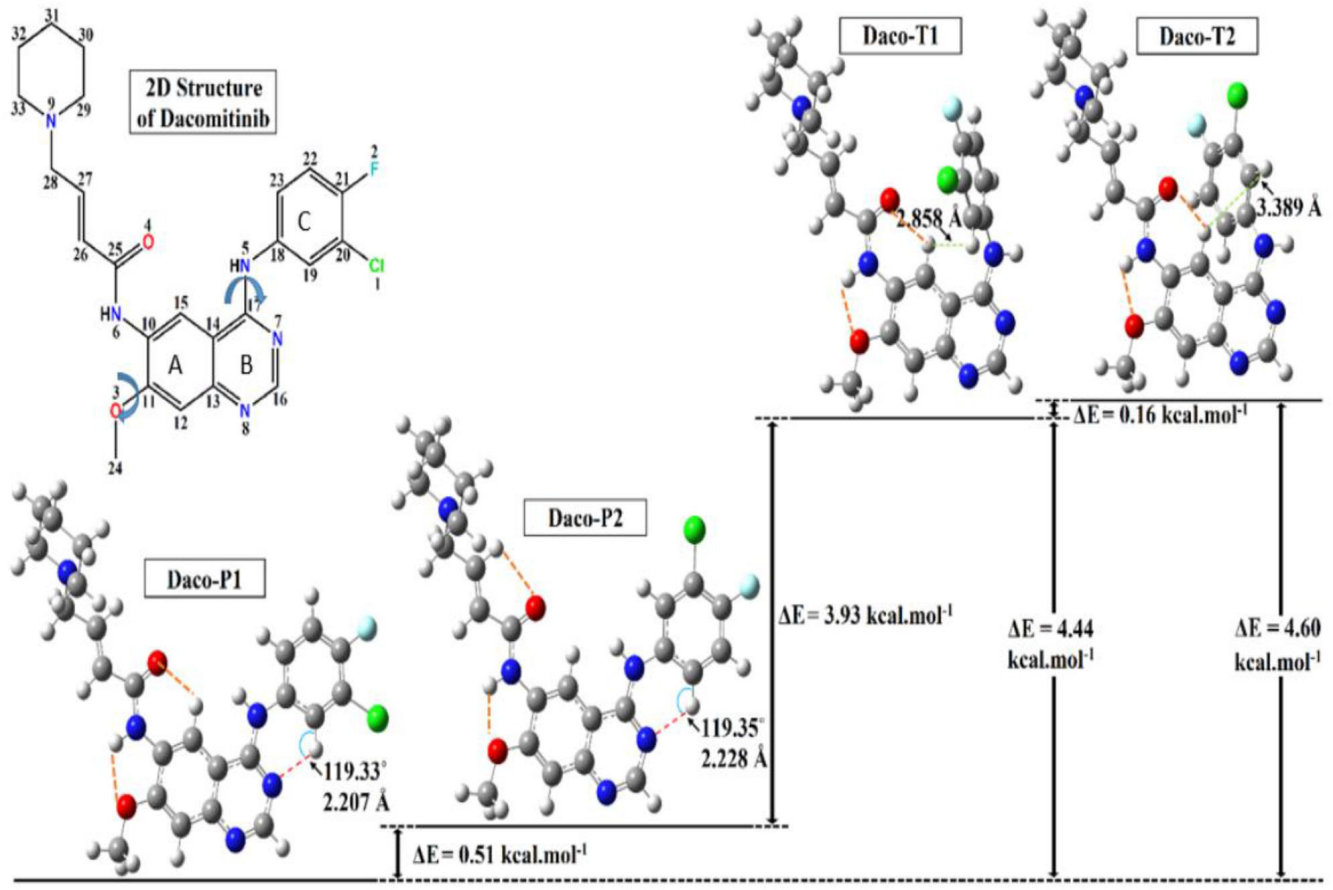
The optimized three-dimensional (3D) structures of the Dacomitinib conformers obtained from the search around the crystal structures are given along with their relative energies. In the 3D structures of Dacomitinib conformers, atoms of carbon, hydrogen, nitrogen, oxygen, chlorine, and fluorine are denoted by gray-, white-, blue-, red-, green-, and cyan-colored atoms, respectively. The energy difference between the most table minimum energy structure (Daco-P1) and the crystal structure (1C9) is 91.15 kcal·mol^−1^.

Figure 3 displays the obtained local minimum structures of Dacomitinib obtained from calculations in isolation. Daco-P1 is evidently the most energetically stable, i.e., global, minimum structure. The other local minimum structures such as Daco-P2, Daco-T1, and Daco-T2 are 0.51, 4.44, and 4.60 kcal mol^−1^, respectively, above the Dac-P1 conformer. Table 1 compares some selective structural parameters of the crystal structures (1C9, 4I23, and 4I24) in Figure 1 and the calculated structures of Dacomitinib in isolation in Figure 3. Table S1 in Supplementary Materials contains the Cartesian coordinates of the four conformers. Figure S1 provides the 3D crystal structure (1C9) and four theoretically obtained Dacomitinib conformers.

As can be seen in Table 1, almost all geometric parameters except for the one highlighted (i.e., the flexible regions and their HB) are very close in values. In particular, the Daco-P1 and Daco-P2 structures exhibit large similarities to the 1C9 crystal structure and the root mean square deviation (RMSD) is smaller than 0.62, which is nearly half the RMSD of Daco-T1 (1.144) and Daco-T2 (1.631). In addition, the dipole moment of the crystal structure is calculated as 11.967 D, which is in close agreement to 12.721 D for Daco-P1 (12.721 D) and Daco-P2 (11.132 D). When in isolation, the “planar” Daco-P1 and Daco-P2 are more stable than the twist conformer pairs. The planar drug (ligand) conformers in dilute solution engage with stronger intramolecular HBs (see Table 1), which contribute to a reduction in the energy. That is, the atoms in these regions are able to form pentagon or hexagon loops through intramolecular hydrogen bonding (HB). For example, the H at the left-hand side forms intramolecular HB of O or N at the right-hand side to close the loop of Hex-1 of H-C(15)-C(10)-N(6)-C(25)-O(4), Hex-2 of H-C(19)-C(18)-N(5)-C(17)-N(7), and Pent H-N(6)-C(10)-C(11)-O(3). The planar structures Daco-P1 and Daco-P2 enhances the intramolecular HB and therefore stabilizes the structures. The three HB of C(15)H···O(4), N(6)H···O(3), and C(19)H···N(7) of Daco-P1 and Daco-P1 are stronger than Daco-T1 and Daco-T2 as their H···O(N) distances are shorter.

The crystal structures are confined structures, which most likely influence the bond angles. The most significant differences of the crystal structures of Dacomitinib are two of the three flexible regions, Hex-2 of H-C(19)-C(18)-N(5)-C(17)-N(7) and Pent H-N(6)-C(10)-C(11)-O(3). In the pair of related dihedral angles C(12)-C(11)-O(3)-C(24) and C(19)-C(18)-N(5)-C(17), the former controls the relevant orientation of the methoxy group to the quinazoline ring and the latter controls the 3-chloro-4-fluoroaniline ring (Ring C) and the quinazoline ring (Rings A and B). As indicated in Table 1, this pair of dihedral angles deviates from planarity (0° or 180°) in the crystal structures (1C9, 4I24, and 4I23) due to confinement in a crystal or a complex. When in isolation, this pair of dihedral angles adopt planar configuration (0° or 180°) (except for the twist conformers) in order to form strong intramolecular HB to reduce the energy as much as possible (Steiner, 2002). As a result, the free Daco-PI conformer in the DMSO solution is more stable than the crystal structure in the same solution by ~9 kcal·mol^−1^.

### Spectral Signatures of Dacomitinib Conformers in the UV-Vis Absorption Spectra

**Figure 4:**
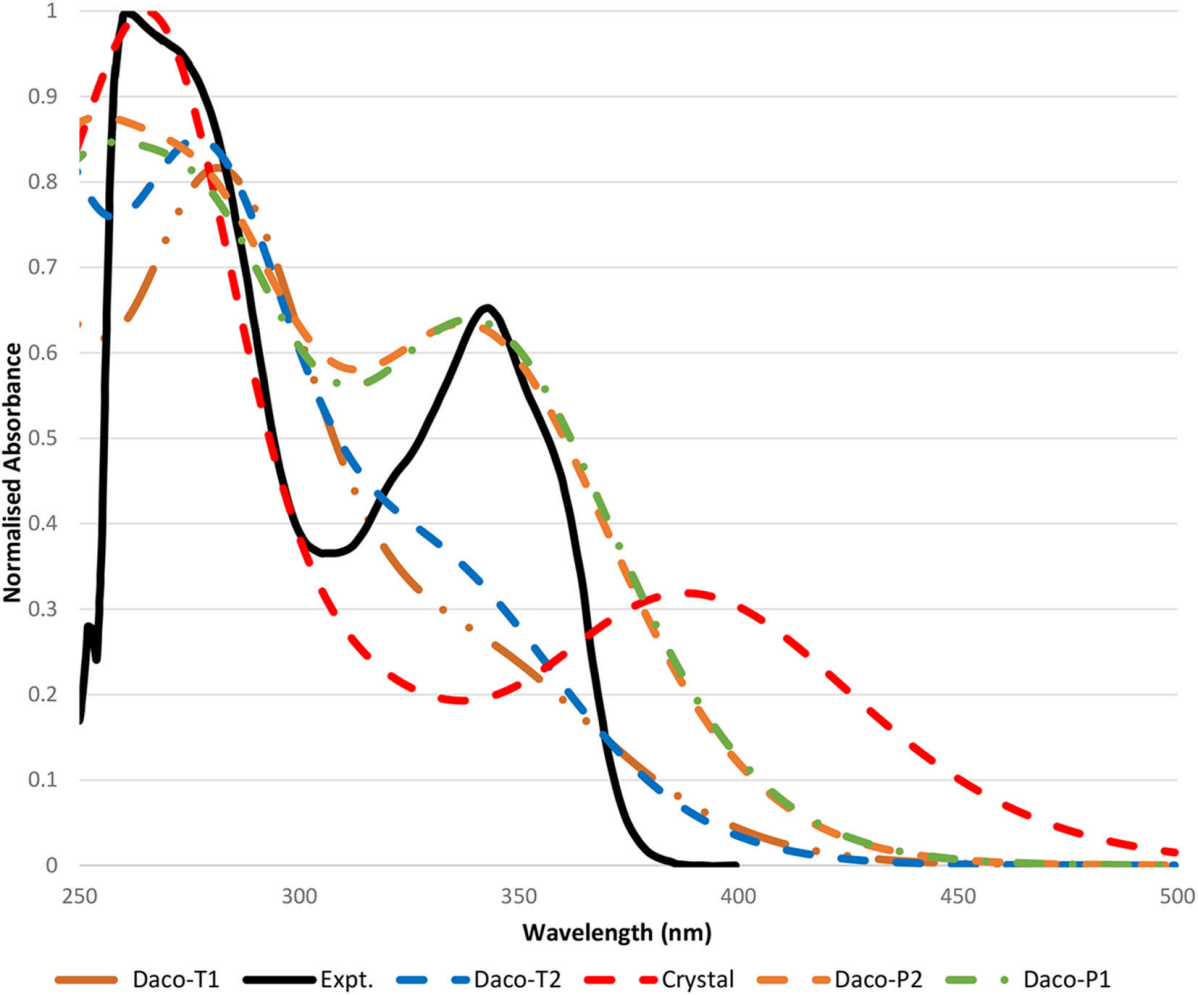
Comparison of the measured UV-Vis absorption spectra of Dacomitinib in the DMSO spectrum (solid black line) with that calculated (colored) in the same solvent. All spectra including both the calculated and the measured are normalized to their own individual maximum absorbances. Please note that the wavelength cutoff due to solvent is 250 nm, and the maximum absorbances of some Dacomitinib conformers lie in the region smaller than 250 nm.

Figure 4 compares the calculated UV-Vis spectra of the Daco conformers with the measurement in DMSO solution. Our previous studies on this class of drugs, such as AG-1478 (Khattab et al., 2016), revealed that the TD-DFT method with the B3LYP/6-311+G(d,p) method produced UV-Vis spectra of the drug in excellent agreement with the measurements, which is in agreement with the present study. The calculated UV-Vis spectra of Daco-P1 using B3LYP/6-311+G(d,p) reproduce the maximum UV-Vis transition of the drug at 345.18 nm, which is in excellent agreement with the measurement of 343 nm in the same solvent of DMSO. While the CAM-B3LYP/6-311+G(d,p) method produces the same transition at 302.74 nm which is approximately a −40-nm shift from the measurement. As a result, B3LYP/6-311+G(d,p) is employed to produce the UV-Vis spectra for other Daco drugs. The resultant UV-Vis spectra are compared in Figure S2 of the Supplementary Materials.

In Figure 4, the experimental UV-Vis spectrum in the region of 250–450 nm of the Dacomitinib drug in DMSO is compared with the calculated UV-Vis absorption spectra of the four conformers of Dacomitinib as well as the crystal structure (1C9 without further optimization) under the same conditions. The experimental UV-Vis spectrum exhibits two major bands, Band A at 261 nm and Band B at 343 nm; both bands (black) have shoulders at 275 nm of Band A and shoulders at 320 and 358 nm at both sides of Band B. UV-Vis spectra, measured at different Dacomitinib concentrations, are given in Figure S3 of the Supplementary Materials.

Inspection of the simulated UV-Vis spectra (see Figure 4) revealed that the planar conformers (i.e., Daco-P1 and Daco-P2) are more likely the dominant Dacomitinib conformations in solution, over the twisted conformers of Daco-T1 and Daco-T2. The simulated UV-Vis spectra of the planar pair of Daco-P1 and Daco-P2 agree better with the measured spectrum, exhibiting two bands at about 280 and 345 nm; the UV-Vis spectrum of the crystal structure (1C9) is the next with two major bands at 262.22 and 390.1 nm. The major HOMO–LUMO transition at 390.1 nm shifts 47 nm from the measured 343 nm. The twisted dacomitinib conformers display one major band at ~283.27 (278.75) nm for Daco-T1 (Daco-T2) and a shoulder band at ~344.03 nm (336.75 nm) for Daco-T1 (Daco-T2). Although the shoulder band above 300 nm stems from the HOMO–LUMO transition, the strength of the band is not significant.

The ratio of the peak intensities at 275 and 343 nm of the experimental UV-Vis spectrum (peak ratio of 1.44) has a close match with the calculated spectra of the planar conformers (peak ratio of 1.29 and 1.33 for Daco-P1 and Daco-P2, respectively), rather than the crystal structure of ~3.0, and the twisted conformers with a peak ratio of 2.99 and 2.64 for Daco-T1 and Daco-T2, respectively. This suggests that in solution the planar conformers are predominant, which is supported by the calculated Boltzmann distribution at the experimental temperature of 293.15 K. That is, the drug in isolation is predominately populated by Daco-P1 (51.08%) and Daco-P2 (48.91%) but the twisted conformers Daco-T2 and Daco-T1 can be neglected. When in isolation including in solution, the Dacomitinib ligand does not take the same conformation as it is in the crystal phase.

**Figure 5:**
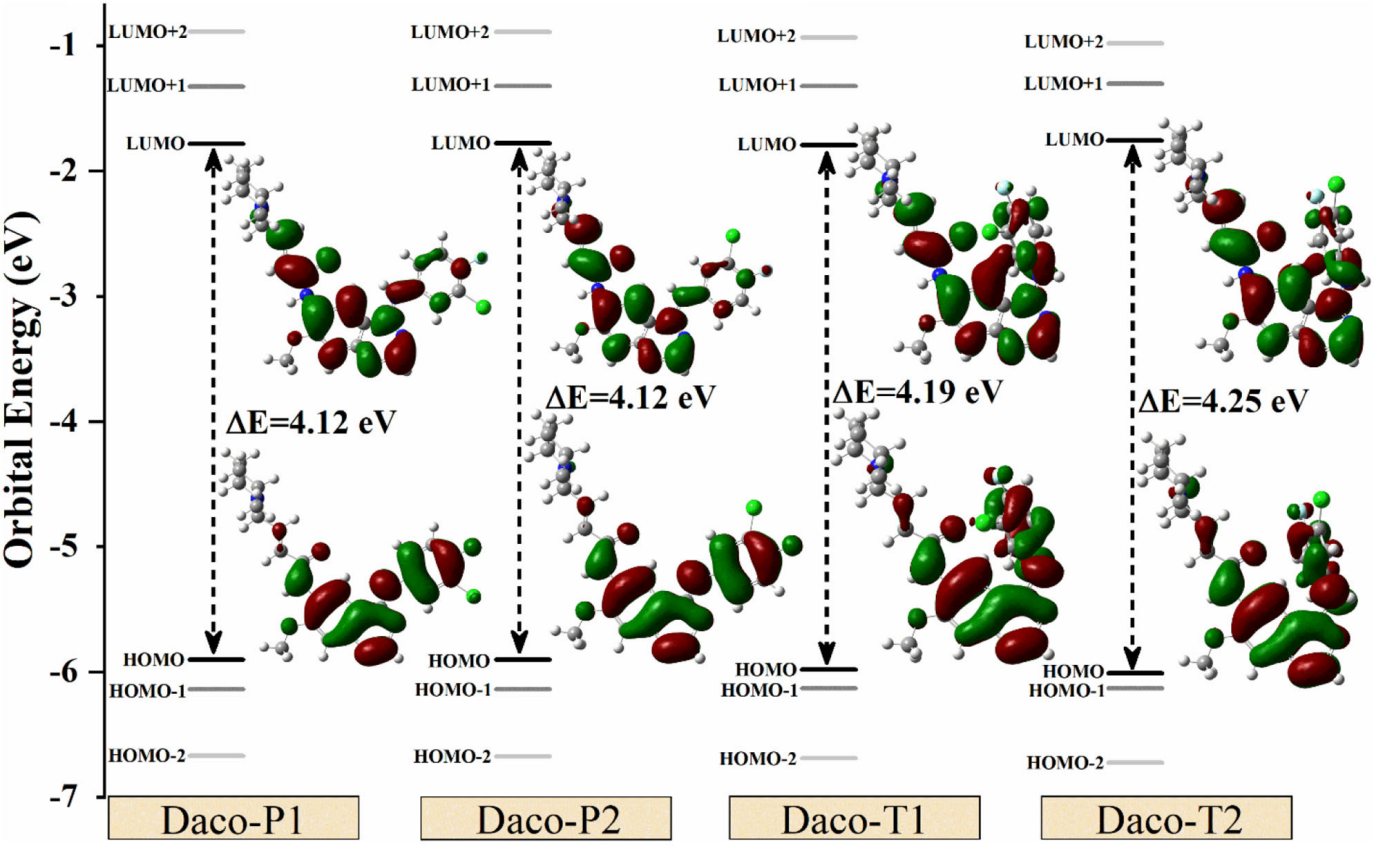
Energy gaps (in eV) among different energy levels (from HOMO-2 to LUMO+2) and HOMO–LUMO orbital charge densities for Dacomitinib conformers in DMSO at the B3LYP/6-311G(d,p) level of theory. The positive and negative electron densities are shown in green and red, respectively.

Figure 5 shows the frontier orbital diagrams ranging from HOMO-3 to LUMO+3 of the low-energy-lying conformers, Daco-P1, Daco-P2, Daco-T1, and Daco-T2, calculated in DMSO solution, along with their corresponding orbital density distributions of HOMO and LUMO. Considering subtle differences in energy gap, Daco-P1 is the lowest-energy configuration and hence most stable structure. The conformers have a small energy difference in HOMO → LUMO energy gap (4.12 eV, 4.12 eV, 4.19 eV, and 4.25 eV for Daco-P1, Daco-P2, Daco-T1, and Daco-T2, respectively). All conformers exhibit a C_1_ point group symmetry including the “planar” Daco-P1 and Daco-P2. As a result, the HOMO–LUMO energy gap or the H → L transitions will be dominant (>96% see Table S2). The HOMO → LUMO gap contributes the major band of UV-Vis (λ > 300 nm in Figure 3), and the planar conformers have slightly smaller HOMO → LUMO gaps and therefore larger λ in the UV-Vis spectra compared with the twisted ones (see Figure 3). The probability of the transitions of electrons increases with the decrease in the energy gap (Δε) between the occupied orbitals (HOMO-*n*) and the virtual orbitals (LUMO+*m*).

Figure 5 also provides the orbitals of HOMO and LUMO for all conformers obtained in isolation. Two apparent features of the frontier orbitals are noticeable: firstly, the orbital electron density of HOMOs and LUMOs concentrates on the aromatic quinazoline and aniline rings through the –N_(5)_H– linker rather than on the piperidine side chain. The planar structure of Daco-P1 and Daco-P2 helps delocalize the HOMO electrons in the conjugate system, whereas the HOMOs of Daco-T1 and Daco-T2 push the HOMO electrons to diffuse into the piperidine side chain through the other –N_(6)_H– bridge. Secondly, the LUMOs of the 3-chloro-4-fluoroaniline ring (Ring C) of Dacomitinib are less populated and the electrons diffuse through the –N_(6)_H– linker toward the piperidine side chain in the Dacomitinib conformers. Folding the 3-chloro-4-fluoroaniline ring (Ring C) over toward the quinazoline ring (Rings A and B) in Daco-T1 and Daco-T2 helps the LUMO electron density to concentrate in the –N_(6)_H– bridge region.

### Electronic Structures of the Dacomitinib Conformers

**Figure 6:**
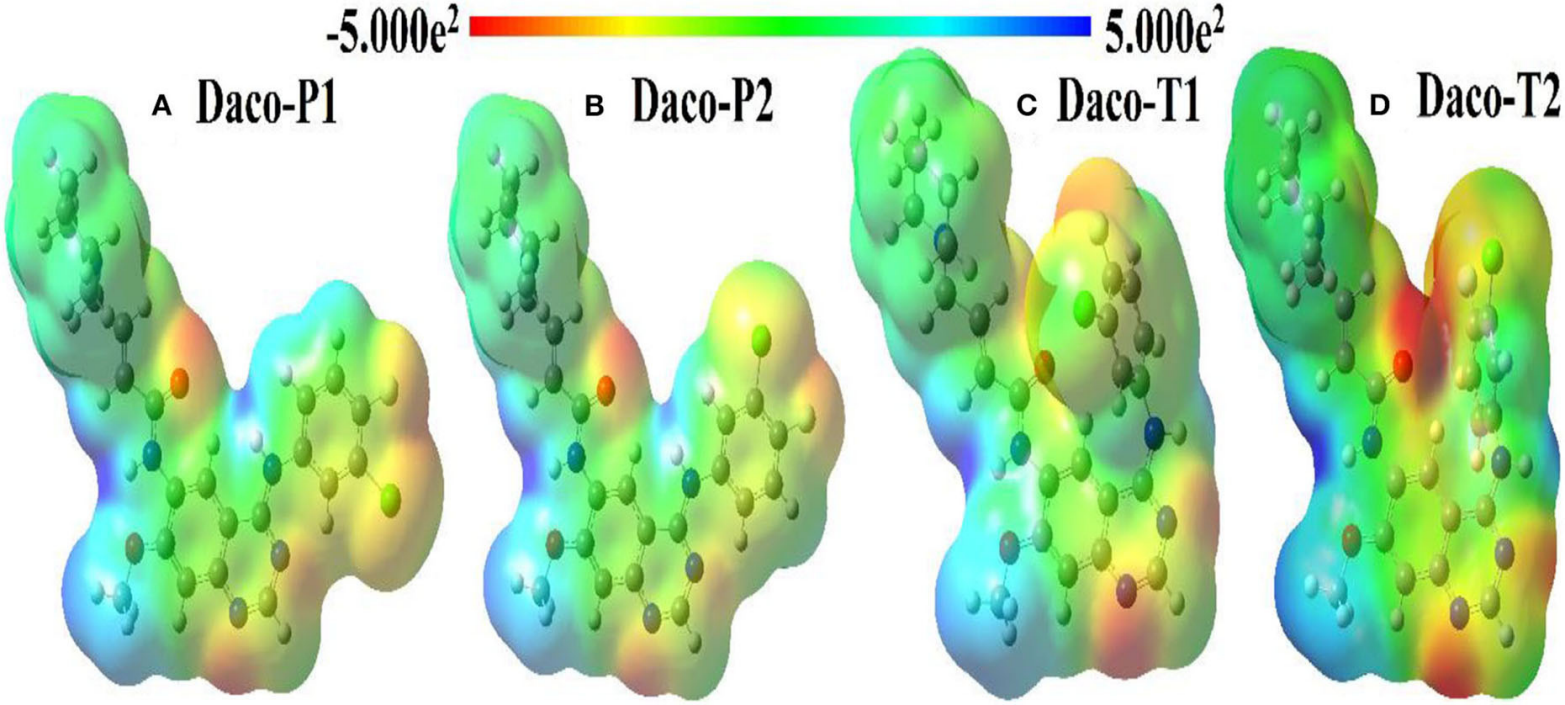
Comparative molecular electrostatic potential (MEP) maps for four conformers of Dacomitinib: **(A)** Daco-P1, **(B)** Daco-P2, **(C)** Daco-T1, and **(D)** Daco-T2 in DMSO solution.

Molecular electrostatic potentials (MEP) of a drug determine approximately the shape of the drug. Shape complementarity is important in drug–target interactions, and so a less stable conformer of a drug can sometimes be more potent if it has the right shape (Chen and Wang, 2009). Hence, it is crucial to realize the differences in electronic structures and shapes among the conformers, which may explain their characteristics like drug potency. In Figure 6, the molecular electrostatic potential (MEP) portrays the distribution of electron density of four conformers along with the scale of the color spectrum having minimum (negative extreme) and maximum (positive extreme) electrostatic potential energy ends marked by red and blue colors, respectively. Overall, in the four conformers, multiple electron donating spots (red) can be identified, which are dominated by the large electronegative atoms such as F, Cl, O, and N as shown in Figure 6. For example, the electron density is withdrawn from the methoxy (O_(3)_-C_(24)_H_3_) group by the quinazoline ring indicated by a light blue color. The electron density of piperidine containing the crotonamide Michael acceptor on C_(10)_ in general is neither high nor low except the amide group (HN_(6)_-C_(25)_O_(4)_). In this amide group, high electron density is observed over a lone-pair electron containing O_(4)_ and the electron less populated in N_(6)_, which are represented as intense red protrusion and deep blue notch, respectively.

The MEPs of planar Daco-P1 and Daco-P2 depict that they both have similar electron density distributions other than the orientation of the yellowish protrusion from the chlorine atom. In contrast to the planar conformers, the twisted ones (Daco-T1 and Daco-T2) have a significant difference in electron density indicated by comparative intense red and blue colors because of their packed structure. A drop in electron density over the N_(6)_ atom and a significant increase in electron localization over the regions of O_(4)_, N_(7)_, N_(8)_, and F_(1)_ atoms are noticeable from Daco-P1 to Daco-T2 [refer to Figure 6 from left (a) to right (b)], respectively. Hence, the planar structures have more electron delocalization and thus are more stable compared to the twisted structures. The shape is the most crucial alteration among the four conformers of Dacomitinib—when the 3-chloro-4-fluoroaniline ring rotates over the single C_(17)_-N_(5)_ bond and folds over the quinazoline ring, which makes the nitrogen atoms (i.e., N_(7)_ and N_(8)_) of the core ring in twisted structures more reactive to form a bond in space, as the MEP map indicates the electrostatic potential of the molecular surface, and it can help in the prediction of intermolecular electrostatic bond formation such as hydrogen bond (an electrostatic force) (Andree and Aakeröy, 2018). The MEP of Daco-P1 and Daco-P2 clearly indicates a possibility of intermolecular hydrogen bond formation between the N_(7)_ atom (high negative potential reflected as reddish MEP surface) and closely aligned hydrogen atom at C_(19)_ (relatively positive potential reflected as a greenish MEP surface).

**Figure 7:**
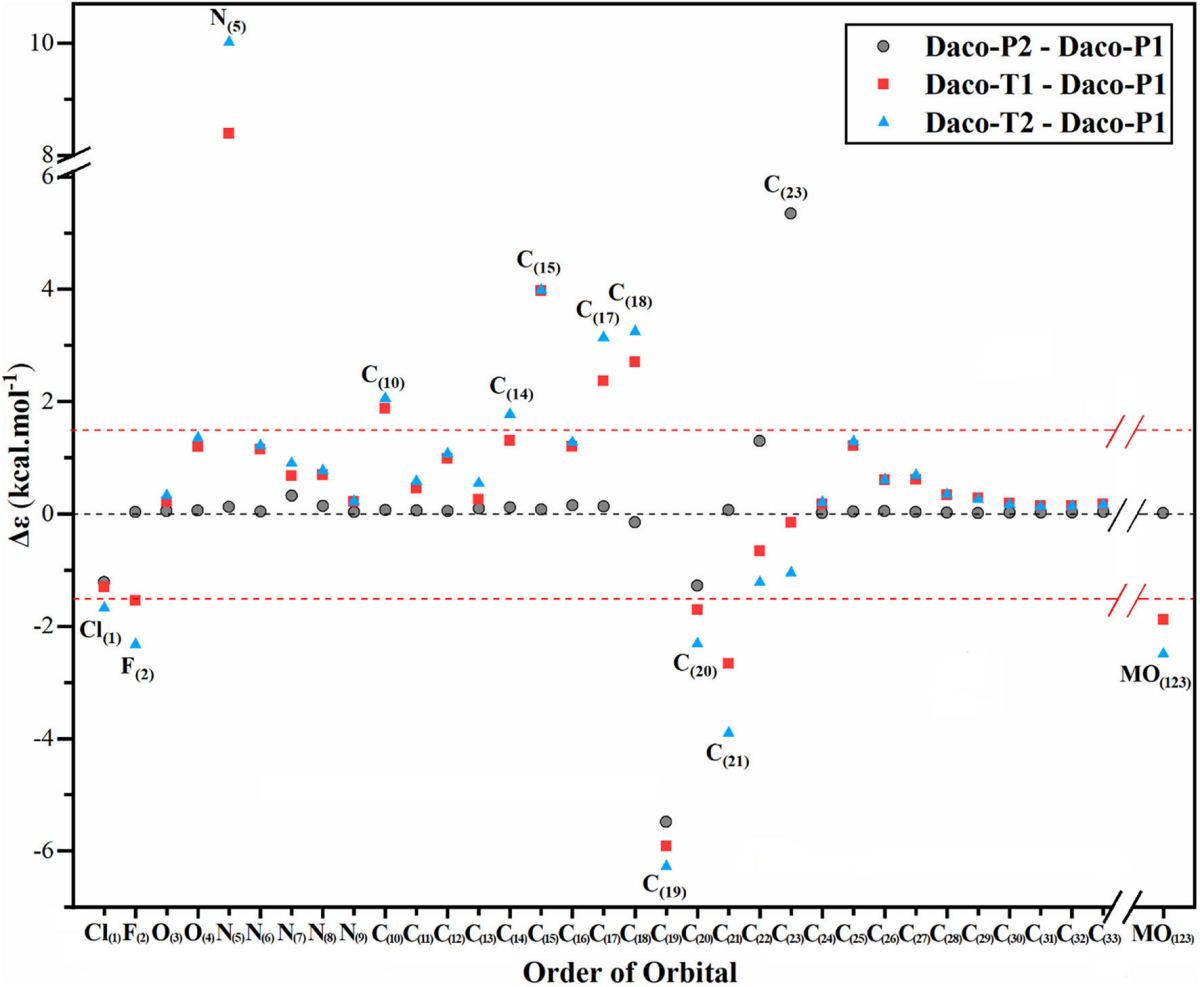
Excess orbital energy spectrum (EOES) of Dacomitinib in DMSO at the B3LYP/6-311G** level of theory: Δε_*i*_ = $\varepsilon_{i}^{lm}$-ε_i_^*gm*^, where $\varepsilon_{i}^{lm}$ = *i*th orbital energy of a local minimum conformer (i.e., Daco-P2, Daco-T1, or Daco-T2) and ε_i_^*gm*^ = *i*th orbital energy of a global minimum conformer, Daco-P1. The X axis is the orbital order from the innermost orbital as Cl_(1)_ to the HOMO as MO_(123)_. The valence orbitals from MO_(34)_ to MO_(122)_ are omitted for simplicity.

Another electronic property which helps to identify specific atoms of the drug conformers is the identification of responsible atomic sites of the drug conformers. The experimental UV-Vis spectrum implies that more than one such Dacomitinib conformer may exist in the DMSO solution. Recent X-ray photoemission spectroscopy (XPS) studies have revealed that conformers experience core electron energy changes (Islam et al., 2018). The excess orbital energy spectrum (EOES) (Islam and Wang, 2015) was therefore introduced to reveal the orbital response to the conformational changes from the reference (which is often the global minimum structure) using a simple method. It is quite helpful in the study of AG-1478 drug conformers (Khattab et al., 2016). The EOES in Figure 6 are three spectra of excess orbital energies Δε_*i*_ = $\varepsilon_{i}^{lm}$-ε_i_^*gm*^, in which $\varepsilon_{i}^{lm}$ is the orbital energies of a conformer and ε_i_^*gm*^ is the orbital energies of the reference which is usually the most stable conformer (Daco-P1). In Figure 7, only atom-specific core 1s energies are considered. The gray solid circles are the excess core 1s energies of Daco-P2 with respect to Daco-P1. The red squares represent the excess core 1s energies of Daco-T1 with respect to Daco-P1, and the blue triangle is the excess core 1s energies of Daco-T2 with respect to Daco-P1. If the excess core 1s orbital energies Δε_*i*_ are outside of the red horizontal dashed lines at a cutoff, for example, ±1.75 kcal mol^−1^ in Figure 6, the conformational impact is significant on that atomic site. As a result, using the core 1s EOES, one can identify the atomic sites which respond significantly to the conformational changes from the most stable conformer (Daco-P1).

In its ground electronic state, Dacomitinib (C_24_H_25_ClFN_5_O_2_) has a total of 123 doubly occupied orbitals, which consists of 33 1s core orbitals. While most of the core 1s EOES (Δε_*i*_ = $\varepsilon_{i}^{lm}$-ε_i_^*gm*^) of Dacomitinib conformers in Figure 7 distribute within the red horizontal dashed lines at ±1.75 kcal mol^−1^ (i.e., a cutoff), a few points, however, locate outside of the two parallel red dash lines. This suggests that most of the atoms in conformer Daco-P2 do not change their energies apparently (those within the parallel red dash lines), and a few atoms, which are local to the particular bond rotation and breaking or formation of hydrogen bonding in conformer Daco-P2, respond to the conformational changes significantly. As shown by the gray solid circles in Figure 6, the most significant core 1s electron-energy changes between Daco-P2 with respect to Daco-P1 are C_(19)_1s and C_(23)_1s, as this pair of carbons, C_(19)_ and C_(23)_, switch their roles in Daco-P1 and in Daco-P2. In Daco-P1, atom C_(19)_ is involved in hydrogen bonding C_(19)_ ···HN_(7)_, and atom C_(23)_ does not. However, in Daco-P2, the hydrogen bonding becomes C_(23)_ ···HN_(7)_, and atom C_(19)_ is not involved in hydrogen bonding.

The EOES shows that in the planar conformer pair of Daco-P1 and Daco-P2, only a small group of atoms associate with apparent core 1s energy changes. In the EOES of the Daco-T1 and Daco-P1 pair (red squares in Figure 7), more atoms are disturbed with significant changes. The most significant atomic 1s energy changes (outside of the parallel red dash line cutoff) are Cl_(1)_1s, F_(2)_1s, N_(5)_1s, C_(10)_1s, C_(15)_1s, C_(17)_1s, C_(18)_1s, C_(20)_1s, and C_(21)_1s. However, the core 1s electron energy changes of Daco-T2 with respect to Daco-P1 (blue triangles) are Cl_(1)_1s, F_(2)_1s, N_(5)_1s, C_(10)_1s, C_(14)_1s, C_(15)_1s, C_(17)_1s, C_(18)_1s, C_(20)_1s, and C_(21)_1s. All of the atomic sites affected significantly due to Daco-T1 and Daco-T2 with respect to Daco-P1 are local to either the halogen atoms, C_(20)_-Cl and C_(21)_-F, or the local atomic site strained with the C_(17)_-N_(5)_ bond rotation, that is, the chain formed by C_(15)_-C_(14)_-C_(17)_-N_(5)_-C_(18)_.

In solution, evidently, multiple conformers of Dacomitinib present and planar conformers are predominant. However, none of our conformers in solution are identical with the previously reported crystal structure (Gajiwala et al., 2013) of Dacomitinib in the complex with its cognate kinase domain. Hence, it is possible that the flexible Dacomitinib changes its conformation when binding with the proteins or confining in crystals, in addition to different phases of the compound.

## Conclusion

In this work, we have used the TD-DFT method to explore the molecular geometry, structural features, molecular properties, and UV-Vis absorption spectra of TKI Dacomitinib in the DMSO solution. Potential energy scans locate two stable planar conformers (i.e., Daco-P1 and Daco-P2) and two less stable twisted conformers (i.e., Daco-T1 and Daco-T2), which all possess lower total energy in isolation than the crystal Dacomitinib structure by over 8 kcal·mol^−1^. The Daco-P1 and Daco-P2 conformers may engage with intramolecular hydrogen bonding, which are C_(19)_-H…N_(7)_, and C_(23)_-H…N_(7)_, respectively, forming a hexagon and pentagon structures to lock the flexibility, hereby contributing to stabilization of the planar conformation. The simulated UV-Vis absorption spectrum of Dacomitinib generated from the contribution of the planar conformers closely reproduces the two measured spectral bands at 275 and 343 nm. In particular, the core 1s energy EOES reveals the atomic site-specific changes of the conformers with respect to Daco-P1. The identified local atomic sites between other conformers and Daco-P1 and Daco-P2 may contribute to the drug potency study. This is because the drug potency significantly depends on the shape/conformation of a drug molecule, in which the flexibility of the ligand and its ability to dock and bind with protein are also important, in addition to their energy. However, an accurate quantum mechanical study on drug/ligand conformers in isolation provides necessary reference information for the ability to form complexes with proteins (Yuan et al., 2017). The crystal structure clearly shows large similarities with Daco-P, the only major difference being the twisting around the N5-C17 bond, which is line with our previous study on a related TKI (Khattab et al., 2016). One potential application of this investigation is to extend the study to identification of the conformer(s) when binding with proteins using molecular dynamic studies, which may contribute to reveal the side effects of the Dacomitinib class of drugs.

## Data Availability Statement

All datasets generated for this study are included in the article/Supplementary Material.

## Author Contributions

MK conducted the experiments, carried out the computational calculations, and writing the manuscript. FB assisted in computational calculations. AC and FW designed and supervised the research. The corresponding authors edited and revised the manuscript.

## Conflict of Interest

The authors declare that the research was conducted in the absence of any commercial or financial relationships that could be construed as a potential conflict of interest.
