# Supplementary material for: Deducing the Conformational Properties of a Tyrosine Kinase Inhibitor in Solution by Optical Spectroscopy and Computational Chemistry
Source: Front Chem. 2020 Jul 28;8:596. doi: 10.3389/fchem.2020.00596 (PMC7399232; doi:10.3389/fchem.2020.00596)
Supplement: Supplementary file 1 [file Data_Sheet_1.docx]

**SUPPORTING INFORMATION**

**Title: Deducing the conformational properties of a tyrosine kinase inhibitor in solution by optical spectroscopy and computational chemistry**

**Author:** Md. Lutful Kabir*^a^*, Frederick Backler*^b^*, Andrew H.A. Clayton*^*a^* and Feng Wang*^*b^*

*^a^Optical Sciences Centre & Department of Physics and Astronomy,*

*Faculty of Science, Engineering and Technology, Swinburne University of Technology, Melbourne, Victoria 3122, Australia. E-mail: aclayton@swin.edu.au*

*^b^Centre for Translatonal Atomaterials & Department of Chemistry and Biotechnology,*

*Faculty of Science, Engineering and Technology, Swinburne University of Technology, Melbourne, Vic. 3122, Australia, E-mail: fwang@swin.edu.au*

*^*^Corresponding authors:* [*aclayton@swin.edu.au*](mailto:aclayton@swin.edu.au)

[*fwang@swin.edu.au*](mailto:fwang@swin.edu.au)

**Figure S1** Crystal structure of Dacomitinib (<https://pubchem.ncbi.nlm.nih.gov/compound/Dacomitinib#section=Crystal-Structures>, 1C9) and optimized structures of Dacomitinib conformers.


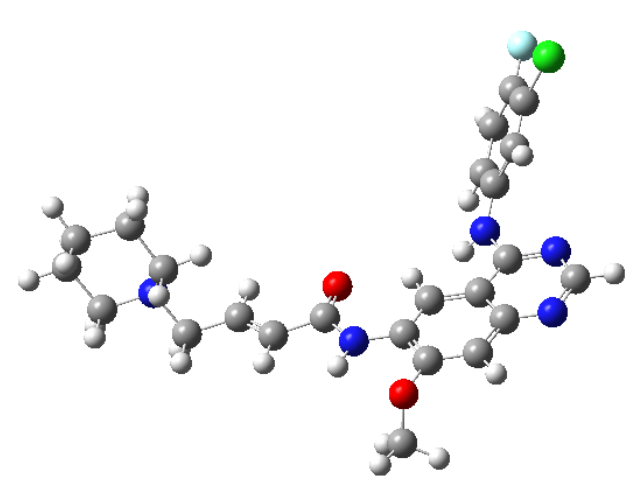

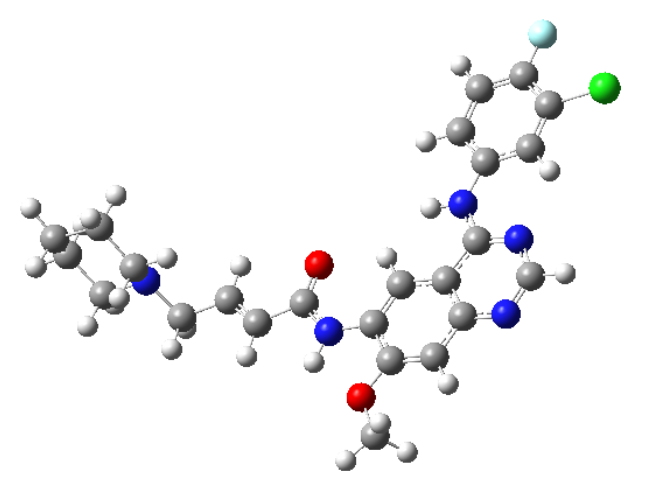

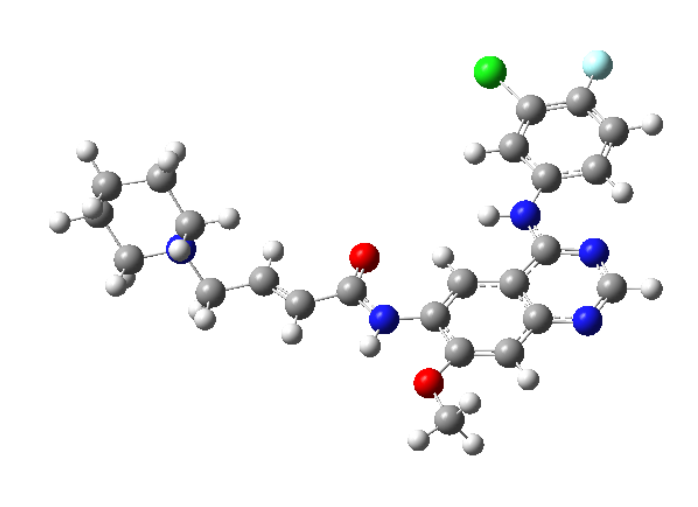


Crystal structure of Daco Daco-P1 Daco-P2


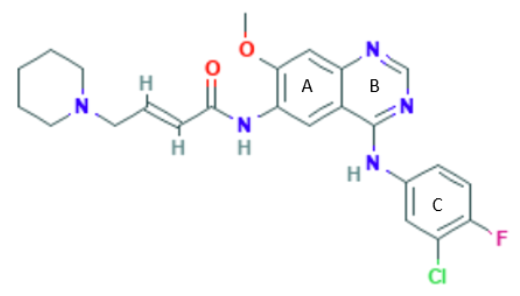

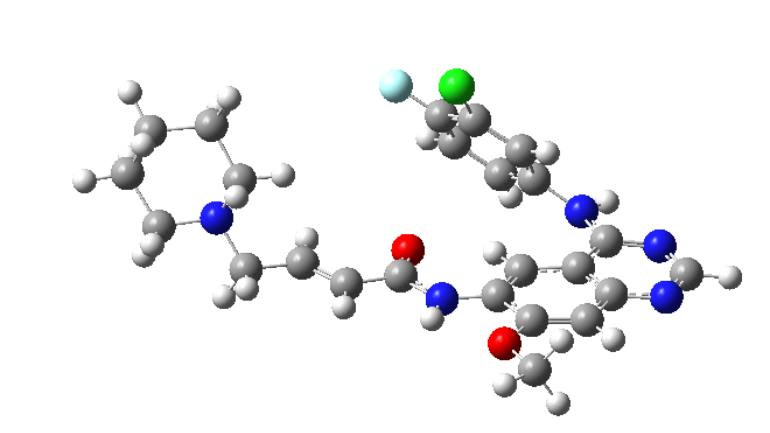

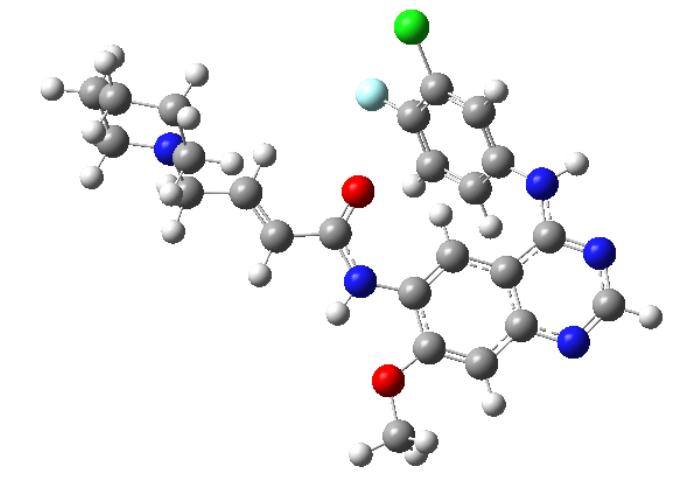


Daco-T1 Daco-T2

**Figure S2** Comparison of the UV-vis spectra of Daco-P1 using TD-DFT method and the B3LYP/6-311+G(d,p) and long range B3LYP/6-311+G(d,p) methods. The calculations are performed on the most powerful supercomputer in Australia, Raijin, which is a 2 petaflop supercomputer provided by Fujitsu (<https://nci.org.au/our-services/supercomputing>).

DFT functional: B3LYP CAM-B3LYP

λ_max_ (Cal) 345.18 nm 302.74 nm

λ_max_ (expt) 343 nm 343 nm

CPU (Raijin) 6 hours 20 minutes 36.9 seconds 7 hours 7 minutes 12.3 seconds


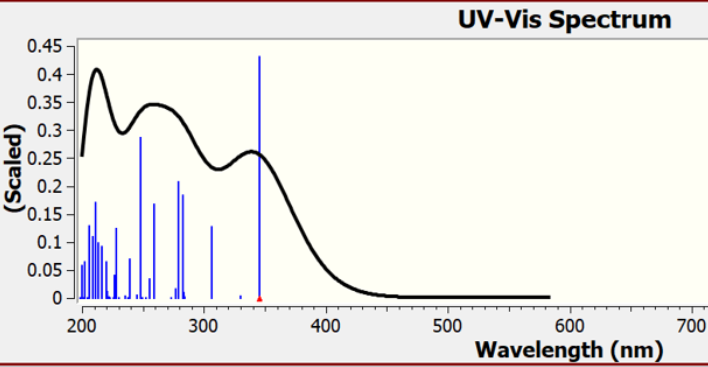

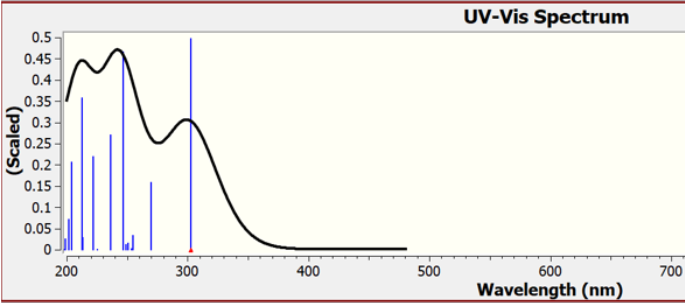


**Figure S3** The measured absorption spectra of Dacomitinib in DMSO solutions with different concentrations at room temperature. The X-axis ranges from 200 nm to 400 nm.


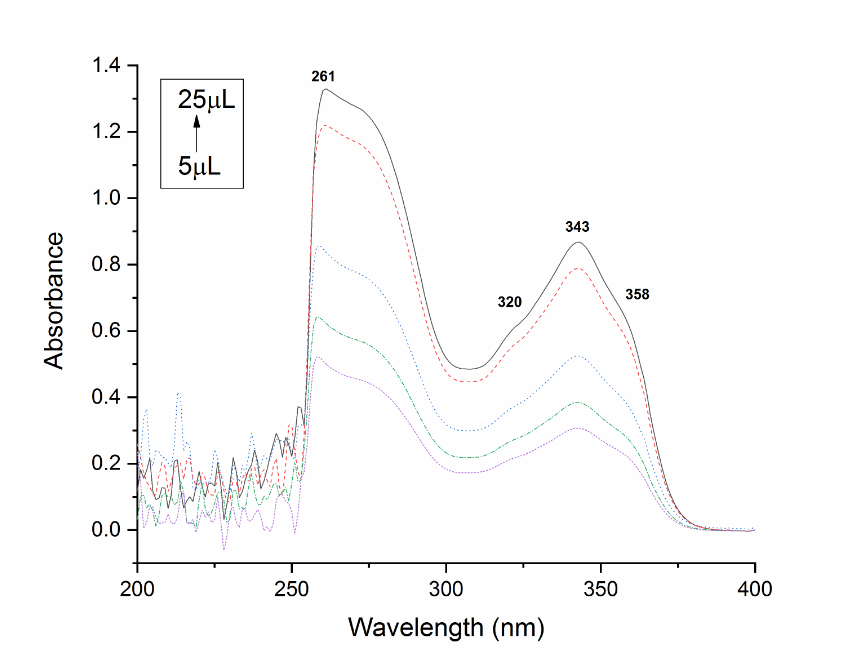


**Table S1** Cartesian Coordinates of Dacominitinib Conformers obtained by geometry re-optimization

using the B3LYP/6-311+G** model of the four local minimum structures of PES (Å).

| Dacomitinib P-1 | | | | Dacomitinib P-2 | | | |
| --- | --- | --- | --- | --- | --- | --- | --- |
| C | 0.46444 | 2.319012 | -0.15625 | C | 0.205424 | 2.535605 | -0.15915 |
| C | 0.207221 | 3.70996 | 0.033116 | C | -0.03569 | 3.933282 | -0.00089 |
| C | -1.06551 | 4.161373 | 0.225038 | C | -1.30201 | 4.402566 | 0.190831 |
| C | -2.14384 | 3.257215 | 0.239433 | C | -2.38966 | 3.510519 | 0.235286 |
| C | -1.89811 | 1.889293 | 0.055969 | C | -2.15997 | 2.136322 | 0.0807 |
| C | -0.57859 | 1.436462 | -0.14222 | C | -0.84676 | 1.664952 | -0.11608 |
| C | -4.35987 | 2.866579 | 0.43774 | C | -4.60721 | 3.147096 | 0.464902 |
| C | -3.05123 | 1.038427 | 0.087859 | C | -3.32175 | 1.298533 | 0.140558 |
| C | -3.80629 | -1.35031 | -0.0933 | C | -4.09699 | -1.08897 | 0.025604 |
| C | -5.17706 | -1.19488 | 0.075761 | C | -3.57389 | -2.37226 | -0.12649 |
| C | -5.99764 | -2.30777 | 0.043001 | C | -4.40252 | -3.4723 | -0.11572 |
| C | -5.47072 | -3.56596 | -0.15489 | C | -5.76464 | -3.30354 | 0.048084 |
| C | -4.11331 | -3.73186 | -0.32302 | C | -6.29368 | -2.04478 | 0.199432 |
| C | -3.28687 | -2.6303 | -0.29223 | C | -5.47037 | -0.93234 | 0.18953 |
| C | 1.154235 | 5.887627 | 0.186795 | C | 0.935396 | 6.10365 | 0.094705 |
| C | 2.335024 | 0.740075 | -0.5572 | C | 2.054411 | 0.927515 | -0.5434 |
| C | 3.807062 | 0.752794 | -0.71137 | C | 3.525323 | 0.918795 | -0.70787 |
| C | 4.479314 | -0.36334 | -0.94323 | C | 4.180654 | -0.2092 | -0.93075 |
| C | 5.952464 | -0.44635 | -1.15542 | C | 5.650841 | -0.3149 | -1.15295 |
| C | 6.506888 | -1.28178 | 1.032221 | C | 6.206333 | -1.14899 | 1.035015 |
| C | 7.911394 | -1.79907 | -0.84985 | C | 7.593038 | -1.69234 | -0.85276 |
| C | 7.040035 | -2.46897 | 1.814886 | C | 6.72779 | -2.34037 | 1.819261 |
| C | 8.497263 | -3.00663 | -0.13932 | C | 8.166506 | -2.90515 | -0.14104 |
| C | 8.452924 | -2.82153 | 1.371196 | C | 8.133245 | -2.71372 | 1.368994 |
| Cl | -7.70994 | -2.121 | 0.253081 | Cl | -3.74692 | -5.06556 | -0.30487 |
| F | -6.27827 | -4.6362 | -0.18479 | F | -6.57238 | -4.37416 | 0.059084 |
| H | -1.28241 | 5.206793 | 0.369051 | H | -1.50667 | 5.453287 | 0.312488 |
| H | -0.34073 | 0.398041 | -0.28829 | H | -0.62049 | 0.620813 | -0.23759 |
| H | -5.36869 | 3.233796 | 0.589349 | H | -5.61039 | 3.528285 | 0.619567 |
| H | -1.93503 | -0.59785 | -0.21581 | H | -2.2224 | -0.34994 | -0.14623 |
| H | -5.59473 | -0.21767 | 0.230888 | H | -2.51076 | -2.51783 | -0.25395 |
| H | -3.7162 | -4.72413 | -0.47672 | H | -7.36103 | -1.9373 | 0.325606 |
| H | -2.22197 | -2.76269 | -0.424 | H | -5.88304 | 0.051832 | 0.308157 |
| H | 2.151743 | 6.307651 | 0.1258 | H | 1.936844 | 6.511399 | 0.017247 |
| H | 0.526795 | 6.31337 | -0.59544 | H | 0.306937 | 6.518108 | -0.69277 |
| H | 0.722835 | 6.1031 | 1.163723 | H | 0.513263 | 6.345981 | 1.069388 |
| H | 2.437381 | 2.756288 | -0.31823 | H | 2.18226 | 2.946699 | -0.34243 |
| H | 4.321807 | 1.701854 | -0.62656 | H | 4.053488 | 1.861684 | -0.63853 |
| H | 3.930279 | -1.29571 | -1.00672 | H | 3.618767 | -1.13482 | -0.97907 |
| H | 6.423086 | 0.523204 | -0.92981 | H | 6.137208 | 0.648803 | -0.93585 |
| H | 6.127121 | -0.64426 | -2.2161 | H | 5.814953 | -0.52064 | -2.21384 |
| H | 7.095547 | -0.38187 | 1.282532 | H | 6.808712 | -0.25631 | 1.278378 |
| H | 5.476251 | -1.07582 | 1.317924 | H | 5.180231 | -0.92786 | 1.325739 |
| H | 7.89047 | -1.96764 | -1.92636 | H | 7.56398 | -1.86435 | -1.92854 |
| H | 8.55818 | -0.92265 | -0.67146 | H | 8.252417 | -0.82404 | -0.68095 |
| H | 6.379955 | -3.32354 | 1.651303 | H | 6.055297 | -3.18653 | 1.662656 |
| H | 7.01459 | -2.23909 | 2.880391 | H | 6.711499 | -2.10614 | 2.883995 |
| H | 9.520755 | -3.16449 | -0.48044 | H | 9.18576 | -3.07831 | -0.48735 |
| H | 7.921719 | -3.89213 | -0.41759 | H | 7.577187 | -3.78365 | -0.41262 |
| H | 8.802675 | -3.72035 | 1.879221 | H | 8.473601 | -3.61529 | 1.878527 |
| H | 9.131542 | -2.01227 | 1.654824 | H | 8.824386 | -1.9127 | 1.645691 |
| N | -3.40308 | 3.746471 | 0.433321 | N | -3.64181 | 4.017038 | 0.430287 |
| N | -4.2552 | 1.536213 | 0.27578 | N | -4.51838 | 1.812525 | 0.330607 |
| N | -2.88379 | -0.29934 | -0.07925 | N | -3.1675 | -0.04362 | -0.00229 |
| N | 1.80187 | 1.975162 | -0.34334 | N | 1.537707 | 2.172571 | -0.34723 |
| N | 6.551544 | -1.53194 | -0.4027 | N | 6.239427 | -1.40537 | -0.39913 |
| O | 1.30608 | 4.486755 | 0.002709 | O | 1.071011 | 4.697388 | -0.05829 |
| O | 1.664621 | -0.27995 | -0.61343 | O | 1.371233 | -0.08503 | -0.57763 |
|  |  |  |  |  |  |  |  |

| Dacomitinib T-1 | | | | Dacomitinib T-2 | | | |
| --- | --- | --- | --- | --- | --- | --- | --- |
| C | -1.74745 | 1.739488 | -0.26432 | C | -1.55829 | -1.84038 | 0.15515 |
| C | -2.52741 | 2.802536 | 0.28573 | C | -2.31332 | -3.039 | 0.340915 |
| C | -3.86067 | 2.637365 | 0.516936 | C | -3.64692 | -3.07129 | 0.058914 |
| C | -4.48263 | 1.403774 | 0.238557 | C | -4.30287 | -1.91217 | -0.40113 |
| C | -3.70889 | 0.336894 | -0.25279 | C | -3.57781 | -0.71446 | -0.52639 |
| C | -2.34196 | 0.542882 | -0.53695 | C | -2.1896 | -0.70949 | -0.27249 |
| C | -6.35645 | 0.150596 | 0.106355 | C | -6.15757 | -0.91231 | -1.21535 |
| C | -4.40969 | -0.88791 | -0.46022 | C | -4.32793 | 0.418137 | -0.96027 |
| C | -2.52476 | -2.52795 | -0.58504 | C | -2.83466 | 2.264308 | -0.22913 |
| C | -1.9118 | -2.27331 | 0.633537 | C | -1.86667 | 3.081961 | -0.78899 |
| C | -0.63708 | -2.74364 | 0.871398 | C | -0.92515 | 3.687751 | 0.01991 |
| C | 0.005014 | -3.50671 | -0.08509 | C | -0.94581 | 3.459442 | 1.382187 |
| C | -0.60935 | -3.80041 | -1.28065 | C | -1.90498 | 2.650708 | 1.949625 |
| C | -1.8716 | -3.2995 | -1.53544 | C | -2.85927 | 2.062252 | 1.144098 |
| C | -2.52659 | 5.053676 | 1.056665 | C | -2.24959 | -5.32758 | 0.989943 |
| C | 0.585049 | 1.180669 | -0.89182 | C | 0.752701 | -0.96182 | 0.362586 |
| C | 1.930059 | 1.798587 | -0.8933 | C | 2.102206 | -1.42447 | 0.758403 |
| C | 3.000235 | 1.09998 | -1.23656 | C | 3.136644 | -0.59927 | 0.749533 |
| C | 4.391311 | 1.633113 | -1.28184 | C | 4.522414 | -0.95955 | 1.164051 |
| C | 5.116521 | 0.637965 | 0.786635 | C | 5.419385 | -1.20181 | -1.05574 |
| C | 6.710624 | 1.122216 | -0.94723 | C | 6.858008 | -0.57732 | 0.766648 |
| C | 6.046775 | -0.38128 | 1.420282 | C | 6.388885 | -0.63418 | -2.07719 |
| C | 7.702109 | 0.121614 | -0.37978 | C | 7.883305 | 0.014231 | -0.18489 |
| C | 7.501849 | -0.04921 | 1.119818 | C | 7.815344 | -0.66417 | -1.54639 |
| Cl | 0.165852 | -2.38252 | 2.363583 | Cl | 0.297457 | 4.70701 | -0.66303 |
| F | 1.242042 | -3.96206 | 0.151751 | F | -0.02282 | 4.033438 | 2.163506 |
| H | -4.47392 | 3.434484 | 0.903074 | H | -4.22832 | -3.97258 | 0.161249 |
| H | -1.74006 | -0.23146 | -0.96827 | H | -1.60077 | 0.174202 | -0.41818 |
| H | -7.43208 | 0.05876 | 0.206736 | H | -7.19172 | -0.97311 | -1.53525 |
| H | -4.49771 | -2.751 | -1.12901 | H | -4.42973 | 2.313121 | -1.53723 |
| H | -2.4134 | -1.6944 | 1.392893 | H | -1.83694 | 3.233531 | -1.85718 |
| H | -0.08485 | -4.39941 | -2.00993 | H | -1.90042 | 2.49913 | 3.018597 |
| H | -2.3519 | -3.49647 | -2.48243 | H | -3.62168 | 1.436941 | 1.583413 |
| H | -1.78537 | 5.836884 | 1.167778 | H | -1.49037 | -6.01248 | 1.35008 |
| H | -3.31185 | 5.383425 | 0.377345 | H | -3.0381 | -5.22663 | 1.734893 |
| H | -2.95681 | 4.812653 | 2.028082 | H | -2.6697 | -5.70115 | 0.05677 |
| H | -0.12698 | 2.960227 | -0.21531 | H | 0.101851 | -2.84866 | 0.743429 |
| H | 2.017714 | 2.833367 | -0.58634 | H | 2.220897 | -2.45996 | 1.052412 |
| H | 2.871937 | 0.061389 | -1.51922 | H | 2.978658 | 0.425704 | 0.434307 |
| H | 4.423952 | 2.642475 | -0.84296 | H | 4.595953 | -2.0443 | 1.338265 |
| H | 4.680708 | 1.733791 | -2.33102 | H | 4.725392 | -0.47052 | 2.120218 |
| H | 5.268019 | 1.621787 | 1.264352 | H | 5.626373 | -2.27653 | -0.90996 |
| H | 4.078648 | 0.357607 | 0.960851 | H | 4.397375 | -1.12319 | -1.4236 |
| H | 6.820093 | 1.190274 | -2.02948 | H | 6.871608 | -0.04263 | 1.716253 |
| H | 6.927841 | 2.123145 | -0.53591 | H | 7.123454 | -1.62687 | 0.982001 |
| H | 5.806485 | -1.37049 | 1.024866 | H | 6.101493 | 0.395417 | -2.30083 |
| H | 5.870271 | -0.41076 | 2.495817 | H | 6.308865 | -1.20173 | -3.0047 |
| H | 8.716767 | 0.454778 | -0.59957 | H | 8.878592 | -0.08637 | 0.248863 |
| H | 7.55945 | -0.83808 | -0.88109 | H | 7.684004 | 1.082146 | -0.29681 |
| H | 8.161976 | -0.82393 | 1.510209 | H | 8.497678 | -0.18534 | -2.24888 |
| H | 7.770266 | 0.881746 | 1.626953 | H | 8.140303 | -1.70361 | -1.44691 |
| N | -5.82618 | 1.297908 | 0.425885 | N | -5.61736 | -2.0007 | -0.74369 |
| N | -5.72179 | -0.95111 | -0.31488 | N | -5.58894 | 0.295854 | -1.33312 |
| N | -3.82039 | -2.04911 | -0.87212 | N | -3.79893 | 1.672155 | -1.07964 |
| N | -0.39884 | 2.025476 | -0.47358 | N | -0.19569 | -1.93691 | 0.435796 |
| N | 5.338885 | 0.733906 | -0.65041 | N | 5.513837 | -0.49205 | 0.213553 |
| O | -1.83652 | 3.932937 | 0.520789 | O | -1.59173 | -4.08513 | 0.783436 |
| O | 0.38174 | 0.022374 | -1.22187 | O | 0.517052 | 0.181348 | 0.002111 |

**Table S2** UV-vis spectral peaks calculated using TD-DFT method and the B3LYP/6-311+G** model of the most stable conformer of Daceo-P1 in DMSO solution (200-500 nm)

# UV-Vis Spectrum

# X-Axis: Wavelength (nm)

# Y-Axis: (Scaled)

# Y-Axis2: Oscillator Strength

# Peak information

# X (nm) Y Y2

# 345.1800000000 0.5744000000 9.8212267094

# 329.6900000000 0.0039000000 0.0666831201

# 306.2100000000 0.1683000000 2.8776331044

# 283.9000000000 0.0014000000 0.0239375303

# 283.4000000000 0.0123000000 0.2103083018

# 282.8500000000 0.2442000000 4.1753892104

# 279.0000000000 0.2772000000 4.7396309955

# 276.5300000000 0.0218000000 0.3727415429

# 273.3000000000 0.0001000000 0.0017098236

# 258.8000000000 0.2225000000 3.8043574910

# 255.2300000000 0.0459000000 0.7848090285

# 252.1900000000 0.0007000000 0.0119687651

# 249.3000000000 0.0002000000 0.0034196472

# 247.8900000000 0.3810000000 6.5144278835

# 244.8300000000 0.0056000000 0.0957501211

# 238.9700000000 0.0927000000 1.5850064693

# 237.7800000000 0.0003000000 0.0051294708

# 235.1700000000 0.0051000000 0.0872010032

# 230.6100000000 0.0002000000 0.0034196472

# 227.9100000000 0.1649000000 2.8194991023

# 226.8600000000 0.0538000000 0.9198850922

# 225.6300000000 0.0002000000 0.0034196472

# 222.5500000000 0.0001000000 0.0017098236

# 222.4700000000 0.0013000000 0.0222277067

# 220.6400000000 0.0013000000 0.0222277067

# 220.4600000000 0.0143000000 0.2445047736

# 219.5800000000 0.0847000000 1.4482205820

# 216.4500000000 0.0001000000 0.0017098236

# 216.1800000000 0.1221000000 2.0876946052

# 212.9500000000 0.1302000000 2.2261903161

# 210.9600000000 0.2273000000 3.8864290234

# 208.8600000000 0.1455000000 2.4877933256

# 205.7400000000 0.1710000000 2.9237983414

# 204.6100000000 0.0003000000 0.0051294708

# 202.1400000000 0.0042000000 0.0718125908

# 202.0900000000 0.0858000000 1.4670286415

# 200.9800000000 0.0001000000 0.0017098236

# 200.2500000000 0.0000000000 0.0000000000

# 199.9100000000 0.0778000000 1.3302427542

# 198.2600000000 0.0000000000 0.0000000000
